# Securing tomorrow’s workforce: insights from a Danish survey on factors influencing junior doctors’ specialization in geriatric medicine

**DOI:** 10.1007/s41999-025-01195-y

**Published:** 2025-05-05

**Authors:** Lene Holst Andersen, Emil Lykke Lundgren, Pernille Holm Ellegaard, Siri Aas Smedemark, Karen Andersen-Ranberg

**Affiliations:** 1 Medical Department, Randers Regional Hospital, Randers, Denmark; 2 Institute for Clinical Medicine, Aarhus University, Aarhus, Denmark; 3 Geriatric Department, Hvidovre Hospital, Copenhagen, Denmark; 4 Medical Department, Zealand University Hospital, Koege, Denmark; 5 Geriatric Research Unit, Odense University Hospital, Odense, Denmark; 6 Department of Internal Medicine, Hospital Lillebaelt, Kolding Hospital, Kolding, Denmark; 7 Department of Geriatric Medicine, Odense University Hospital and Geriatric Research Unit, Odense, Denmark; 8 Department of Clinical Research, University of Southern Denmark, Odense, Denmark; 9 Department of Geriatric Medicine, Aarhus University Hospital, Aarhus, Denmark

**Keywords:** Work force recruitment, Geriatric education, Work-life balance, Geriatric specialist training

## Abstract

**Purpose:**

With aging populations, more geriatricians are needed. Despite the demand, filling specialist-training positions remains challenging, and the factors influencing junior doctors’ career choices in Denmark are not well understood. This study aimed to investigate the factors influencing junior doctors’ decisions to choose geriatric specialist training.

**Methods:**

A literature review informed the development of a survey, which included demographic and open-ended questions about prior geriatric training, reasons for pursuing geriatric positions, and perspectives on geriatric specialist training. The survey targeted junior doctors completing their introductory year positions in Internal Medicine and Geriatrics at Danish hospitals from March to September 2023. Qualitative data were analyzed using reflexive thematic analysis.

**Results:**

Out of approximately 130 eligible respondents, 75 provided responses. Among them, 13% (*n* = 10) expressed interest in pursuing a geriatric career, and 44% (*n* = 33) indicated a potential interest. In total, 60% (*n* = 45) reported having received geriatric training during medical school. Thematic analysis generated three themes. 1) Achieving core geriatric medicine competencies: This included receiving geriatric education and practicing in specialized geriatric services, e.g., hospital-at-home. 2) Achieving work-life balance: This appeared particularly important in multi-center training programs or during emergency department rotations. 3) Enhancing recruitment strategies: This involved acknowledging talents, utilizing role models, increasing visibility in medical school curricula, and strengthening the specialty’s professional reputation. Finally, ten recommendations were generated.

**Conclusion:**

Recruiting junior doctors to pursue a career in geriatric medicine may benefit from targeted learning in geriatric competencies, the presence of role models, and ensuring that clinical training practices support work-life balance.

**Graphical Abstract:**

**Supplementary Information:**

The online version contains supplementary material available at 10.1007/s41999-025-01195-y.

## Key summary points

**Aim:**

To investigate factors influencing junior doctor’s choice of geriatric specialist training.

**Findings:**

The study emphasizes the importance of promoting geriatrics as a rewarding career choice through targeted education and hands-on experiences. This includes supervised clinical tasks and managing geriatric services, such as falls clinics or hospital-at-home programs. In addition, fostering a supportive work environment with flexible hours, job satisfaction, and strong mentorship can help attract and retain junior doctors in the field of geriatrics.

**Message:**

To cultivate interest in geriatrics among junior doctors, it is essential to highlight the specialty’s meaningfulness and provide excellent geriatric education starting in medical school. Moreover, it is crucial to ensure a supportive and balanced work environment.

**Supplementary Information:**

The online version contains supplementary material available at 10.1007/s41999-025-01195-y.

## Graphical abstract

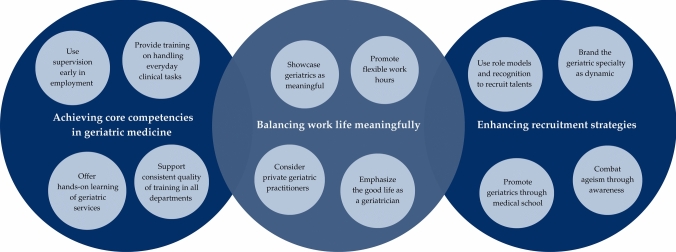

## Introduction

The increasing number of geriatric patients, coupled with shortages of health professionals, has made recruitment in the healthcare sector more crucial than ever [1, 2]. The shortage of geriatric specialists varies across Europe, with uneven distribution and recruitment challenges [3, 4]. In Denmark, geriatricians are concentrated in the capital, with fewer in rural areas, similar to Germany and Spain [3]. While the British Geriatric Society recommends one geriatrician per 500 people aged 85+, Denmark has 181 specialists—one per 800 citizens [5]. Despite the growing demand, the fill rate for physician training positions in geriatrics in Denmark is not keeping pace [6]. The Danish Health Authority’s projection of doctors and specialists forecasts an increase in the number of geriatricians from the current 181 in 2024–497 by 2045 [6]. Between 2019 and 2023, the Danish Health Authority allocated a total of 89 specialist training positions, of which 80 were filled, corresponding to a filling rate of 90% [7]. To address this gap, it is essential to understand the driving factors behind junior doctors’ career choices, which can inform strategies to attract and retain talent in this critical field.

Junior physicians’ career decisions are shaped by both intrinsic and extrinsic factors. Intrinsic factors reflect personal priorities and interests, as well as the professional identity and work/life balance [8]. Extrinsic factors, such as working environmental factors, encompass workforce shortages and geographic location of careers [9–11]. In many European countries, such as Denmark, the salaries of hospitalists are mainly determined through collective bargaining agreements, and to a lesser extent personal wage supplements [12]. As a result, variations in income among Danish hospitalists are minimal and as such, salaries are not considered an extrinsic factor.

Globally, several challenges contribute to low recruitment rates in geriatric medicine, including misconceptions about the specialty, limited exposure during medical school, and perceived lower prestige compared to other fields [13]. Ageism may play a role, as media often depicts aging in a stereotypical way, and the emotionally demanding nature of the specialty, focusing on multimorbidity and end-of-life care, can also deter junior doctors [14, 15].

The recruitment of geriatric specialists faces significant challenges due to demographic changes. This study aims to explore the factors influencing junior doctors’ decisions to choose specialist training in geriatrics. By examining their motivations and preferences, we generated recommendations to improve recruitment strategies and better attract aspiring and talented geriatricians.

## Methods

### Study design and setting

We conducted a cross-sectional study to explore the factors influencing junior doctors’ decisions to enter geriatric specialist training. Our study used a survey with open-ended questions and adhered to the Standards for Reporting Qualitative Research (SRQR) guidelines [16]. A phenomenological approach was employed to gain an in-depth understanding of participants’ lived experiences [17]. The study targeted Danish junior doctors nearing or recently completing an internal medicine introductory year with a focus on geriatric medicine.

### Danish healthcare system and educational system

**Fig. 1:**
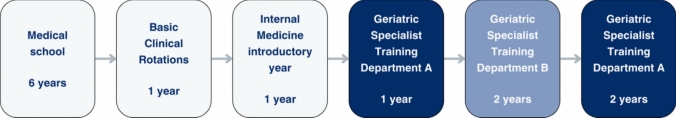
The educational pathway to becoming a geriatrician in Denmark

In the Danish healthcare system, citizens registered in the Civil Registration System with a place of residence in Denmark have access to publicly funded healthcare, including hospital admissions [18]. The system is tax-funded. Figure 1 outlines the educational pathway to becoming a geriatrician, which includes 6 years of medical school, 1-year basic clinical rotations, and an internal medicine introductory year, which qualifies for internal medicine specialist training including geriatrics. While the introductory year primarily focuses on internal medicine, some hospitals offer geriatric-focused training, either in geriatric departments or through supervision by geriatric specialists. Geriatric specialist training then spans 5 years, divided between two locations, which can be up to 170 km apart.

Figure 1 illustrates that becoming a geriatric consultant requires at least 12 years of training. The dark and light blue colors represent Departments A and B, located in separate sites within the specialist training program.

### Survey development

Following a literature review, the Educational Board of the Danish Society of Geriatrics and Danish Early Career Geriatricians developed the survey between January 2023 and March 2023. The survey featured demographic data, ensuring participant anonymity, and open-ended questions to 1) explore participants’ expectations towards the internal medicine introductory year and rating of the geriatric specialty after completing this position and 2) explore factors influencing junior doctor’s decision to choose specialist training in geriatric medicine. Participants could also provide recommendations for the geriatric specialty. The survey is included in Appendix 1.

### Data collection

The survey was distributed from March to September 2023 through Postgraduate Clinical Lecturers in geriatrics, Chief Consultants in internal medicine or geriatric departments, and via social media channels of Danish Geriatric Society and Danish Early Career Geriatricians. Eligible participants included junior doctors in their final stages of their introductory year of specialist training or those who had recently completed it. Annually, most departments in Denmark report the number of medical positions [19]. Using this data, and contacting the departments not listed, we identified approximately 130 eligible doctors. Survey responses were collected using REDCap electronic data capture tools hosted at Aarhus University [20]. Informed consent was obtained from all participants. In accordance with Danish law, survey-based studies that do not involve the collection of sensitive personal data are exempt from formal ethical approval.

### Data analysis

Qualitative data from open-ended questions were analyzed using Braun and Clarke’s six-step reflexive thematic analysis [21, 22]. The research team, consisting of junior doctors in specialist training and geriatric consultants, brought valuable contextual insights but employed a reflexive approach to account for potential biases.

In the first step, data were read several times to ensure familiarization with data. Then data were coded inductively in step two, with researchers (LA, EL, PE, and SS) collaboratively coding two sections of open-ended questions together to ensure quality in coding. Subsequent responses were coded individually by each of the researcher (LA, EL, PE, and SS) after the first ten responses were coded together. In case of uncertainty, the case was discussed among the researchers. In the third step, potential themes were identified. Step four and five included iterative theme review and refinement to create a cohesive narrative. In the final step, quotes were selected for the manuscript. In addition, the ten recommendations were developed through iterative review and synthesis of key findings.

## Results

In total, 75 responses were collected. Of these, 13% (*n* = 10) indicated a definite interest and 44% (*n* = 33) indicated a potential interest in pursuing a geriatric career. Overall, 60% (*n* = 45) recalled having had geriatric training during medical school. Table 1 holds the demographic data.

**Table 1:** Demographics, *n* = 75

Recalled having geriatric teaching during medical school, *n* (%)	
Yes	45 (60,0)
No	24 (32,0)
Not sure	6 (8,0)
Clinical rotations during medical school, *n* (%)	18 (24,0)
Educational region in Denmark, *n* (%)	
East	36 (48,0)
South	19 (25,4)
North and others	20 (26,6)
Introductory year of specialist training, department, *n* (%)	
Geriatric department	34 (45,3)
Internal medicine department	41 (54,6)
Applying for specialist training in geriatric medicine, *n* (%)	
Yes	10 (13,3)
No	32 (42,6)
Maybe	33 (44,0)
Overall perception of the geriatric specialty after introductory year^a^, median (IQR)	8 (7–9)

^a^The overall perception of the geriatric specialty was assessed using a ten-point Likert scale ranging from 1 (very negative) to 10 (very positive)

An association was observed between the medical school attended and the proportion of participants expressing interest in pursuing geriatric specialist training. The university where geriatrics is sparsely taught had no participants who indicated an interest in pursuing geriatric specialist training. Qualitative data analysis generated three themes.

### Achieving core competencies in geriatric medicine

Most junior doctors chose an introductory year in specialist training to enhance internal medicine skills, without necessarily intending to become geriatricians. These skills encompassed handling medical issues including polypharmacy, complex sector transitions, and practical skills, such as ultrasound as a bed-side examination.

“Because I am unsure about which specialty to choose, I thought geriatrics provided an opportunity to work across many specialties.” (Participant 43)

However, many participants did not receive the training they sought for, particularly in areas like ward rounds, medication management, and frailty assessment, and they emphasized the need for more structured teaching and supervision.

“I must admit that I thought there would be more time for teaching us junior doctors. It’s very much “just try it yourself.” Geriatricians have so much knowledge, and I wish there was a higher priority on teaching, so we can be properly trained rather than self-taught.” (Participant 26)

Participants expressed frustration over insufficient training, which discouraged some from pursuing geriatric specialist training.

“We want more education! Teach us how to manage medications, assess falls, evaluate frailty etc. All those things you are experts in, which we read up on ourselves continuously, but honestly would sharpen our interest in geriatrics if we were trained. Make us a priority, rather than just a tool for work.” (Participant 23)

Many participants were not introduced to what life as a geriatric specialist entails. What does the outpatient clinic look like for a specialist? How much can I influence my own day-to-day life as a geriatrician in the future? Several participants recommended showcasing geriatric services, such as falls clinics and hospital-at-home services.

“Understandably, many of the geriatric services were reserved for registrar-level doctors, but since I was never assigned these services and ward rounds were far too busy, there was too little focus and time to learn geriatrics.” (Participant 7)

### Balancing work life meaningfully

#### Tailored work-life balance

Work-life balance was important to most participants, and many also worried about the working conditions after completing specialist training, such as whether heavy shift workloads and overtime would collide with their family life priorities. Several participants called for flexible working hours, e.g., allocated time for research, thus having the departments tailor training positions to their individual needs. Although the survey did not directly inquire about alternative career preferences, several participants spontaneously mentioned that their career considerations often lay between geriatrics and becoming a general practitioner, with working hours and a more flexible working life often drawing them towards general practice.

“It’s about deciding whether I want to be a hospital doctor or a general practitioner. I need a general practice training position to make a better decision. Maybe the working conditions with shifts and workload in general at hospitals are a bit of a downside.” (Participant 44)

Several participants mentioned establishing private geriatric clinics as a reason for preferring the specialty.

“I am enthusiastic about the geriatric specialty. The only thing holding me back is the limited opportunities outside the hospital, such as the chance to work in or open a private practice.” (Participant 40)

Concerns about long commutes in multi-center specialist training programs also deterred interest, particularly for those with family commitments. Some participants associated rural training sites with lower training quality, further discouraging long-distance commuting.

“My assumption might be that the quality of education, you get, is lower in hospitals in more rural areas than in centrally located hospitals, which would make the long commute even more unbearable.” (Participant 35)

#### Working climate and the emergency department

Participants highlighted the working climate as a key factor in choosing a specialty, emphasizing collaboration, uninterrupted time for growth, and a strong sense of departmental solidarity.

“Colleagues and the working climate are more important to me than being able to practise geriatrics, which is otherwise the specialty I am most passionate about.” (Participant 39)

Participants had mixed views on working in emergency departments (EDs). Some found ED work rewarding, particularly when focused on acute geriatrics. However, most felt ED shifts diverted attention from developing specific geriatric competencies and cited them as a reason for not pursuing the specialty. Suggestions for improvement included shorter shifts and a balanced workload.

“If geriatrics did not include the ED […] I would have no doubt about choosing geriatrics as my specialty” (Participant 37).

Participants emphasized the importance of a skilled training environment and suggested improvements to both working and educational settings. Many participants viewed inter-professional collaboration as either a motivation for pursuing an introductory year position or a goal for improving teamwork with therapists and nurses. Recommendations included enhanced training in core geriatric competencies, continuity in work schedules (e.g., consecutive ward round days), and increased supervision in daily clinical practice.

“More quality courses in the introductory year of specialist training that provide insight into what one learns more of in specialist training. So that one not only feels like part of production but also feels valued and educated.” (Participant 32)

#### A meaningful work life

Geriatric medicine was widely viewed as meaningful, with its holistic patient approach and focus on patient-centered care and advocacy for the older patient.

“Geriatrics is such a broad medical specialty; you need to be able to do many things, have a pragmatic approach to treatment, and be willing and ready to adapt treatment to the patient, not necessarily according to guidelines. You need to consider the patient as a whole.” (Participant 75)

Participants highlighted the importance of developing strong communication skills, particularly for difficult conversations, interdisciplinary collaboration, and involving relatives. Palliative care was also seen as a significant and rewarding aspect of the specialty, offering dignity and value to patients in their final stages of life.

“ […] to see the whole person and not just one organ. To give value to older persons and provide a dignified end to life.” (Participant 73)

### Enhancing recruitment strategies

#### Recognition and nudging

Many participants identified active recruitment strategies as key motivational factors for choosing geriatric specialist training. These strategies included involving junior doctors in departmental activities such as administrative tasks, teaching, and interdisciplinary conferences, along with providing recognition.

“Tell your junior doctors what they are good at and why their skills would be valuable in the specialty” (Participant 50)

The department’s responsibility to allow junior doctors to participate in the annual meeting of the national geriatric society and the European Geriatric conference was also noted as measures in active recruitment. If a consultant taps you on the shoulder and says*, “Wouldn’t you like to come to our European conference?”,* this brings great pride and recognition that strengthen the choice of pursuing a career in geriatric medicine. The value of good role models was highlighted as crucial for recruiting doctors to geriatrics. Geriatrics was seen as a master’s apprenticeship, requiring mentors to inspire and guide younger doctors towards a holistic approach.

#### Branding

Several respondents suggested that the Danish Geriatric Society should prioritize branding efforts, focusing on both the geriatric specialty and its patient group. While some expressed concerns about the complexity of the patient population, they also highlighted the potential to frame these challenges as opportunities for growth.

“Believe that the ‘complex’ and challenging patients will eventually not seem daunting but instead be a source of lifelong learning.” (Participant 77)

As part of branding efforts, the participants advocated for combating ageism and addressing prejudices held by colleagues in other specialties toward older patients.

“After my year in a geriatric department, I encounter colleagues who think geriatricians are skilled and competent internal medicine specialists, but there is also prejudice and ageism that diminishes the specialty.” (Participant 64)

Participants encouraged the society to target medical students by providing early exposure to geriatrics. Suggestions included establishing a geriatric student society, prioritizing students during clinical placements, and showcasing the specialty’s breadth, expertise, and holistic approach. In addition, junior doctors called for reforms in medical education to include more geriatric teaching.

“Attempt to engage medical students earlier in their studies—which requires more education in geriatrics. […] We somehow need to change the perception of geriatrics—and it is best to do so early in medical school, in my opinion.” (Participant 64)

## The ten recommendations

Table 2 provides a set of participants’ recommendations aimed at increasing interest in geriatric specialist training.

**Table 2:** Ten recommendations for better recruitment to the geriatric-specialist training

#	Recommendation
1	Promote geriatrics as a meaningful career and show life as a geriatrician
2	Provide geriatric education on handling everyday clinical tasks, such as conducting ward rounds, medical reviews, etc
3	Let junior physicians try geriatric services such as falls clinics and hospital-at-home
4	Create flexible working hours and job positions with room for research, etc
5	Ensure work-life balance and focus on job satisfaction, especially when physicians visit other departments, e.g., the emergency department maintaining a geriatric focus
6	Be aware that the career choice often lies between geriatrics and becoming a general practitioner and consider establishing private clinics for geriatric medicine to provide more opportunities outside of hospital settings
7	Develop and share training programs to ensure consistent quality across peripheral and university hospitals
8	Promote local role models and recruit geriatric talents actively by nudging and recognition
9	Showcase a dynamic and evolving specialty and brand geriatrics to avoid ageism
10	Enhance the impact of geriatric medicine in medical schools and introduce geriatric role models early

## Discussion

This study explored factors influencing junior doctors to choose an educational geriatric position. Key factors and recommendations identified in the thematic analysis include the need for early exposure to geriatrics in medical school, stronger branding of geriatric medicine, and the important impact of role models. The findings emphasized the importance of comprehensive training in core geriatric competencies during the introductory year of specialist training, alongside support for work-life balanced and a positive working climate. These elements collectively provide a strategic framework to enhance interest and proficiency in geriatric medicine.

A significant finding was that many participants could not recall receiving geriatric training during medical school, particularly at universities with minimal exposure. In Denmark, few medical students visit geriatric departments, and the ECTS credits for geriatric courses range from 0.1 to 5.7 out of 360 total ECTS. This limited exposure may be aggravated by a shortage of trained geriatricians and academic faculty to mentor and educate students [13, 23, 24]. This situation persists despite the existence of extensive undergraduate and graduate curricula designed to address geriatric education and care [25–28]. Moreover, medical students often perceive geriatric care as overly complex and show low enthusiasm toward patient-centered care [13, 29]. To address these challenges, integrating geriatrics more prominently and consistently throughout medical education is essential. The Comprehensive Geriatric Assessment (CGA), a cornerstone of geriatric medicine, exemplifies a patient-centered approach, but remains difficult to incorporate into curricula [30, 31]. Defining the geriatric patient and geriatric assessment more clearly might favor recruitment [24]. While we did not assess early-career initiatives, participants emphasized the need to strengthen geriatrics in medical schools. This could be facilitated through student associations or early-career geriatrician initiatives.

Participants emphasized the need to rebrand geriatrics as a dynamic and evolving specialty, as it is often perceived as less prestigious compared to other fields [32]. Branding efforts should highlight the holistic nature of geriatric medicine, opportunities for interdisciplinary collaboration, and the diverse career paths, including research, leadership, and non-hospital roles. By portraying geriatrics as a vibrant and rewarding field, the specialty could attract a new generation of doctors who might otherwise overlook it [28]. Emphasizing roles such as palliative care may further strengthen the appeal. Notably, participants suggested that branding efforts address ageism, a concern affecting both recruitment and patient safety [33]. Ageism "often begins during medical education [34, 35]. Incorporating education on aging and fostering positive, personalized, and equal interactions between generations, particularly through face-to-face engagement could mitigate ageism [33, 36].

Positive role models emerged as a critical factor in shaping interest in geriatrics. Some of the participants pointed out the lack of visible geriatricians as mentors during their training, which contributes to a lack of interest in the specialty. Role models are pivotal in influencing medical students’ and junior physicians’ perceptions and career choices [11, 32]. Establishing local junior geriatric ambassadors and promoting the achievements of geriatricians within departments could foster a more supportive environment that cultivates interest in the field. Nguyen [37] calls for a personal approach to each physician and suggests assessing learner needs, evaluate training outcomes in work settings and offer feedback on their daily practice.

Several participants pursued an introductory year in geriatrics to enhance internal medicine skills, viewing geriatrics as foundational for their medical careers. As such, they expressed frustration when their requests for geriatrics education were unmet. Traditionally, European medical curricula focus on organ-specific conditions, training students to be specialists rather than generalists [26]. With the increasing number of older patients, it is critical to prioritize geriatric education on institutional level, beyond individual departments.

In Denmark, a medical education reform is underway to equip specialists with broader competencies for managing older patients with multiple chronic conditions [38]. This reform aims to ensure that specialists retain generalist skills, fostering cohesive, patient-centered care [38]. Internationally, attention to geriatrics education is growing, particularly due to initiatives, such as the COST PROGRAMMING [39].

Challenges in geriatrics education include the heterogeneous nature of geriatric patients and difficulties adhering strictly to guidelines [13]. Traditionally, geriatrics has required extensive hands-on training, making the influence of role models particularly crucial, as also noted by our study participants. The Geriatric 5Ms framework, gaining attraction in the US and Canada, offers a simplified yet holistic approach to patient care [40]. Its adaptability and common language make it accessible to those outside the field, enhancing its educational value, and might also help students engage with the complexity of the geriatric specialty.

Work-life balance and job satisfaction were key factors influencing the decision to specialize in geriatrics. Participants valued flexible work arrangements, such as part-time positions and opportunities to engage in research. However, many found working in emergency departments problematic, emphasizing the need to improve the work environment and allow for the development of geriatric competencies. Similar recruitment challenges in emergency medicine highlight the importance of addressing “*recruitment red flags*”, such as negative perceptions of work-life balance or the lack of mentorship, to attract more candidates [41]. At the departmental level, participants advocated for proactive strategies, including recognition and nudging, which may positively impact job satisfaction and the work environment.

The distance between training sites was another significant barrier to choosing geriatrics. In Denmark, the migration of younger doctors to urban areas exacerbates healthcare disparities in rural regions, mirroring broader systemic inequities seen in other countries like the UK [42, 43]. Addressing this imbalance requires strategies to redistribute the workforce, such as financial incentives, targeted recruitment campaigns, and improved rural working conditions [44]. Insights from primary care, where similar challenges have been addressed successfully, could inform these efforts [45, 46]. In addition, tailored onboarding schemes for new practitioners could improve integration and ensure they feel supported within the specialty.

This study highlights several factors that deter doctors from pursuing a career in geriatrics. A perceived lack of structured supervision, teaching, and onboarding contributed to the sense that geriatrics training prioritizes service delivery over education. The absence of procedural skills in geriatrics was also noted as a drawback. Overall, work-life balance and hospital workloads are considered a red flag, with many doctors favoring primary care physicians. Other challenges included the predominance of emergency departments in geriatrics training, thus limiting exposure to geriatrics-specific functions such as outpatient care. Concerns regarding career progression and professional development were also evident, with limited exposure to research opportunities.

This study has several limitations. First, findings rely on self-reported data, which may introduce response and selection bias, as individuals with more positive views on geriatrics might be more inclined to participate. However, the inclusion of respondents who ultimately chose not to pursue geriatric medicine suggest that selection bias was minimal. The sample size, though adequate for the purpose, may not fully represent the diverse experiences and perspectives of all junior doctors interested in geriatrics. In addition, this survey was conducted in Denmark, and this might limit the generalizability to other European countries. However, we believe that our findings can serve as a model for adaptation and application across Europe, ultimately enhancing recruitment strategies for future geriatricians.

Another potential limitation is the influence of researcher biases, as the authors’ backgrounds as geriatricians and trainees may have shaped the interpretation of the data, particularly in valuing aspects related to geriatric care. However, this background allows us to more profoundly comprehend the stakes involved, given our direct immersion in the field. Finally, the use of open-ended survey questions limits the depth of insight into participants’ perceptions. Another limitation is the absence of data on gender and age. These data were not collected to preserve participant anonymity and encourage open and honest responses. While this approach aimed to foster candid and varied responses, including critical reviews, it limited our ability to analyze potential associations between characteristics and interest in geriatric specialist training. Future research should include interview studies to better understand the factors influencing career decisions.

## Conclusion

This study explored the factors influencing the choice of geriatric specialist training among 75 junior doctors in Denmark. The findings underscore the need for targeted interventions to improve the visibility, training, and perception of geriatrics. Strategies, such as improving educational experiences, fostering positive role models, and offering flexible, rewarding career pathways are essential to addressing recruitment and retention challenges. These measures are crucial not only for the future of the specialty but also to meet the growing healthcare needs of an aging population.

## Supplementary Information

Below is the link to the electronic supplementary material.

Supplementary file 1 (DOCX 16 KB)

## Data Availability

Data are available from the corresponding author upon request.
