# Supplementary material for: Securing tomorrow’s workforce: insights from a Danish survey on factors influencing junior doctors’ specialization in geriatric medicine
Source: Eur Geriatr Med. 2025 May 5;16(3):739–47. doi: 10.1007/s41999-025-01195-y (PMC12174228; doi:10.1007/s41999-025-01195-y)
Supplement: Supplementary file 1 — (DOCX 16 KB) [file 41999_2025_1195_MOESM1_ESM.docx]

**Appendix 1** Questions after completing an introductory position in geriatrics

| Did you receive education in geriatrics during medical school? | o Yes  o No o Don’t know |
| --- | --- |
| Did you attend a geriatrics clinic during medical school? | o Yes  o No |
| Where did you complete your medical education? | o Aalborg University o Aarhus University o University of Southern Denmark o University of Copenhagen o Other |
| Where did you complete your introductory position? | o Geriatrics department o Internal medicine department with geriatrics |
| Did you have an employment in another internal medicine department before your current position | o Yes  o No |
| Did you have an introductory position/employment in general practice before your current position | o Yes  o No |
| What was the reason for your interest in an introductory position in geriatrics? | Free text |
| What were your expectations for your introductory position in geriatrics? | Free text |
| Are you planning to apply for specialist training in geriatrics? | o Yes  o No o Maybe |
| IF YES, MAYBE: What do you think would attract more people to apply for specialist training in geriatrics? | Free text |
| IF NO: What would it take for you to apply for specialist training in geriatrics? | Free text |
| What is your view of the geriatrics specialty after completing an introductory position in geriatrics? | 10-item Likert scale:  Very negative \| Neutral \| Very positive |
| What is the reason for your response to the previous question? | Free text |
| Do you have any advice for the geriatrics specialty or geriatrics departments going forward? | Free text |
